# Histoplasmosis in the Republic of Congo dominated by African histoplasmosis, *Histoplasma capsulatum* var. *duboisii*

**DOI:** 10.1371/journal.pntd.0009318

**Published:** 2021-05-06

**Authors:** Fructueux Modeste Amona, David W. Denning, Donatien Moukassa, Michel Develoux, Christophe Hennequin

**Affiliations:** 1 Faculty of Health Sciences, Marien Ngouabi University, Brazzaville, Republic of Congo; 2 Research Center and Study of Infectious and Tropical Pathologies, Oyo, Republic of Congo; 3 The University of Manchester and Manchester Academic Health Science Centre, Manchester, United Kingdom; 4 Assistance Publique-Hôpitaux de Paris, Hôpital St Antoine, Service de Parasitologie-Mycologie, Paris, France; 5 Sorbonne Université, Inserm, Centre de Recherche Saint-Antoine, CRSA, AP-HP, Hôpital Saint-Antoine, Service de Parasitologie-Mycologie, Paris, France; Yale University School of Medicine, UNITED STATES

## Abstract

The Republic of Congo (RoC) is one of the African countries with the most histoplasmosis cases reported. This review summarizes the current status regarding epidemiology, diagnostic tools, and treatment of histoplasmosis in the RoC. A computerized search was performed from online databases Medline, PubMed, HINARI, and Google Scholar to collect literature on histoplasmosis in the RoC. We found 57 cases of histoplasmosis diagnosed between 1954 and 2019, corresponding to an incidence rate of 1–3 cases each year without significant impact of the AIDS epidemic in the country. Of the 57 cases, 54 (94.7%) were cases of *Histoplasma capsulatum* var. *duboisii (Hcd)* infection, African histoplasmosis. Three cases (5.3%) of *Histoplasma capsulatum* var. *capsulatum* infection were recorded, but all were acquired outside in the RoC. The patients’ ages ranged between 13 months to 60 years. An equal number of cases were observed in adults in the third or fourth decades (*n =* 14; 24.6%) and in children aged ≤15 years. Skin lesions (46.3%), lymph nodes (37%), and bone lesions (26%) were the most frequent clinical presentations. Most diagnoses were based on histopathology and distinctive large yeast forms seen in tissue. Amphotericin B (AmB) was first line therapy in 65% of the cases and itraconazole (25%) for maintenance therapy. The occurrence of African histoplasmosis in apparently normal children raises the possibility that African histoplasmosis is linked to environmental fungal exposure.

## Author summary

The Republic of Congo (RoC) is one of the African countries with the most histoplasmosis cases reported. Here, we review what is published regarding epidemiology, diagnostic tools, and treatment of histoplasmosis in the RoC. We found 57 cases of histoplasmosis diagnosed between 1954 and 2019, corresponding to an incidence rate of 1–3 cases each year. There was no relationship with the increasing rates of HIV in the country. Most of the 57 cases we found (95%) were cases of African histoplasmosis caused by the fungus *Histoplasma capsulatum* var. *duboisii*. Those affected varied in age from 13 months to 60 years, with equal numbers observed in adults in their third or fourth decades and in children (approximately 25% each). Skin lesions (46%), enlarged lymph nodes (37%), and bone lesions (26%) were the most frequent clinical presentations. The diagnosis was usually based on histopathology with distinctive large yeast forms seen in tissue. Amphotericin B (AmB) and itraconazole (25%) were used for therapy. African histoplasmosis in apparently normal children raises the possibility that this disease is linked to environmental fungal exposure.

## Introduction

Histoplasmosis is an endemic mycosis due to a dimorphic fungus named *Histoplasma capsulatum* [1]. Its distribution is worldwide [2,3], but Africa is unique in regard to this infection [4] as 2 clinical entities coexist due to *Histoplasma capsulatum* var. *capsulatum* (*Hcc*) and *Histoplasma capsulatum* var. *duboisii* (*Hcd*), the cause of “African Histoplasmosis.” Indeed, while the geographic distribution of the former encompasses South and North America, Asia, and Africa, the latter has only been reported in patients living or having lived in Africa [4,5].

While the HIV/AIDS pandemic and the increased use of immunosuppressive agents clearly demonstrated the opportunistic behavior of *Hcc*. Indeed, there were reports of cases in previously ‘‘non-endemic areas” revealing the global distribution of histoplasmosis in Africa [5] and throughout the world [2,3,6,7]. In countries where patients have limited access to diagnostic testing and antiretroviral therapies (ARTs), histoplasmosis is probably an important cause of mortality in persons living with HIV/AIDS. Histoplasmosis, particularly *Hcd*, has not been adopted by the World Health Organisation as a neglected tropical disease (NTD) (https://www.who.int/neglected_diseases/diseases/en/) despite early consideration [8] and was also rejected by G-Finder, despite a formal application (Global action fund for fungal infections, 2016).

In the Republic of Congo (RoC), the health epidemiological profile is characterized by the predominance of infectious diseases, mainly malaria, TB, and HIV/AIDS infections [9]. Many studies have been published since the description of the first case of *Hcd* infection reported in 1954 in the RoC [10]. However, the real problem of histoplasmosis in the RoC is underestimated, as the available information on this disease comes from case reports, case series, or old reviews published in the literature.

The knowledge gap of the local epidemiology of this disease is a serious limitation for effective infection control and treatment approaches. Therefore, we performed in this study an exhaustive review of the published cases from 1950 to 2019, including some cases published in non-English language, to provide the current status of histoplasmosis in the RoC regarding the epidemiology, diagnostic tools, and treatment.

## Methods

### Ethics statement

This analysis did not deal with individual patient data but with published data, which does not require regulatory approval.

### Search strategy and eligibility criteria

Computerized literature searches for publications on histoplasmosis cases or series in the RoC were performed using online databases Medline, HINARI, and PubMed. The search engine used the key words and the detailed medical subject heading (MeSH) terms to identify all published papers: “Histoplasmosis,” “Congo,” “Brazzaville,” “HIV/AIDS,” “diagnosis,” “epidemiology,” “Africa,” or “Sub-Saharan Africa.” The Boolean operators “AND” and “OR” were used to combine 2 or 3 terms. All included studies were cases and series reports (*n =* 57) originating from the RoC. Although peer review articles published in the English language were included, most were in French language. No date limitation or any other search criteria were applied to avoid missing papers published in the RoC. Systematically, we searched Google Scholar and gray literature papers regarding the subject, to supplement Medline and PubMed searches. Some cases reports were excluded from some parts of our analysis due to the lack of information related to sex, age, clinical presentation, and treatment.

Further review of relevant individual cited references identified additional cases published in French-language journals such as the Journal of Medical Mycology (formerly Journal de Mycologie Médicale) et Armée, Médecine d’Afrique Noire, Médecine Tropicale, or Bulletin de la Société de Pathologie Exotique.

## Results

### Cases described from the Republic of Congo

#### Epidemiology

**Fig 1:**
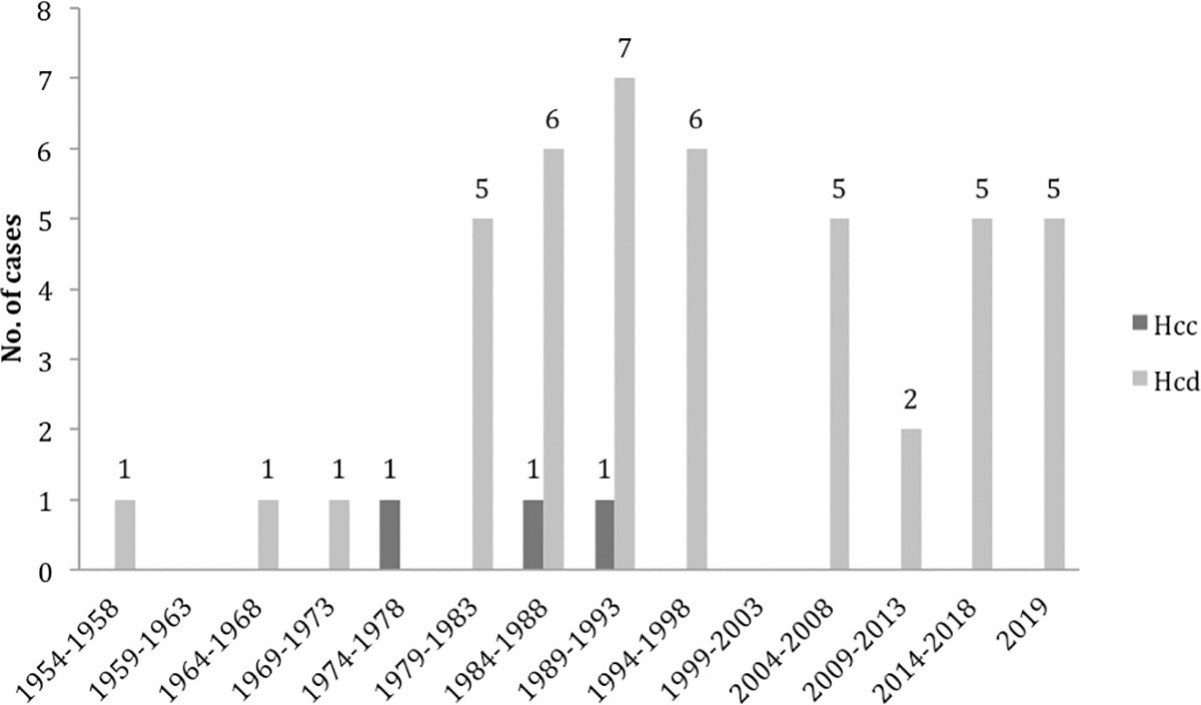
Cases of histoplasmosis reported from the Republic of Congo (1954–2019). Light grey bars: *H*. *capsulatum* var *duboisii*. Dark grey bars: *H*. *capsulatum* var *capsulatum*.

Our exhaustive literature search revealed a total of 57 diagnosed cases of histoplasmosis, including 54 cases (94.7%) of *Hcd* infection and 3 *Hcc*, were reported in the RoC between 1954 and 2019 (Tables 1 and 2). All were reported as single case report but 1 series of 11 cases reported by Carme and colleagues in 1990 [11]. The documentation of these cases varies from one study to the other and also from one case to another within the same study. For example, in 13 and 14 cases, sex or age, and clinical presentation (12 cases) were lacking. Also, the treatment of patients was not specified in 17 cases. Thus, those cases were excluded from some parts of our analysis. This total of cases represents a mean incidence of 1 to 3 cases each year, without significant change over the years, notably in regard to the AIDS epidemic (Fig 1).

**Table 1:** Description of 54 cases of *Hcd* infection in the RoC.

Age/ Sex	No. of cases	Years	Origin areas	Occupation	HIV infection	Site of infection	Disseminated form	Treatment	Posology	Outcome	References
-/M	1	1954	Urban	NS	NT	Brain, Skin (Subcutaneous)	Yes	Terramycine, pentamidine	1.5 g/day for 1 month	Good general condition. Chronic evolution of the disease	Audebaud et al. [10]
15/F	1	1970	Rural	NS	NT	Skin	NS	NS	NS	NS	Destombes et al. [12]
NS	1	1967	NS	NS	NT	Skin	NS	NS	NS	NS	Renoirte et al. [13]
44/M	1	1981	Rural	Farmer	NT	Skin	No	AmB	0.6 to 1 mg/kg to 1 mg/kg in 1 day over 2. Given as an infusion	Favorable outcome	Carme et al. [14]
6/F	1	1982	Rural	Pupil	NT	Skin	No				
36/M	1	1983	Rural	Farmer	NT	Skin, mucosa	No				
13/F	1	1983	Rural	Pupil	NT	Mucosa, Bone	No				
25/M	1	1983	Rural	Unemployed	NT	Skin	No				
45/M	1	1984	Rural	NS	NT	Skin	No				
11/M	1	1984	Urban	NS	NT	Skin, Bone	Yes	AmB	1.6 g/24 h every 2 days	Favorable outcome	Griffet et al. [15]
26/F	1	1985	NS	Unemployed	NT	Skin	No	AmB	0.6 to 1 mg/kg to 1 mg/kg in 1 day over 2. Given as an infusion	Favorable clinical response	Carme et al. [14]
27/M	1	1986	NS	Farmer	Negative	Skin	No				
2/F	1	1987	NS	-	NT	Skin, Bone, Eye	Yes	AmB			
13/M	1	1988	NS	Pupil	NT	Skin, Bone, Shoulder	Yes	AmB			
17/M	1	1989	NS	Pupil	Negative	Skin	No	AmB			
17/M	1	1989	NS	Pupil	Negative	Skin, mucosa	No				
26/M	1	1990	NS	Student	Positive	Skin	Yes	AmB KETO	0.6 to 1 mg/kg to 1 mg/kg in 1 day over 2. Given as an infusion	Death	
50/M	1	1990	NS	Farmer	Negative	Skin	Yes	AmB KETO	0.6 to 1 mg/kg to 1 mg/kg in 1 day over 2. Given as an infusion	Death	
13/M	1	1991	Rural	NS	NT	Bone	Yes	AmB, Surgery	1.50 g Given as an infusion	Favorable clinical response at months 3. No relapse at 1 year	Moyikoua et al. [16]
NS	11	1990	NS	NS	Positive	NS	Yes	NS	NS	NS	Carme et al. [11]
26/M	1	1992	NS	Student	Positive	Skin (cutaneous and subcutaneous)	Yes	NS	NS	Death	Carme et al. [17]
4/M	1	1995	Urban	-	Negative	Lung, Skin, Bone	Yes	KETO AmB	1/2 tablets/day 0.5 mg/kg × 2 days	Favorable clinical response	Chandenier et al. [18]
20/F	1	1995	Rural	NS	Positive	Skin	Yes	AmB ITRA	1 mg/kg × 2 days 300 mg/days	Death	
44/M	1	1995	Urban	Driver	Positive	Skin	Yes	KETO AmB	600 mg/days for 2 months and half. 1 mg/kg, 3 days/week. Given as an infusion	Death	
45/M	1	1995	Urban	NS	NT	Skin	-	NS	NS	Death	
41/M	1	1995	Urban	NS	Positive	Skin	Yes	AmB	1mg/kg/week	NS	
32/M	1	1995	Urban	Nurse	Positive	Skin, Bone	Yes	AmB	1mg/kg/week	NS	
NS	1	2004	Urban	NS	Positive	NS	Yes	NS		NS	Ondzotto et al. [19]
17/M	1	2004	Urban	NS	Negative	Skin, Bone	Yes	AmB	0.25 mg/kg. Given as intravenous infusion. Increased by 5mg/days to a maximum of 1 mg/kg/day	Favorable clinical response at months 11	N’Golet et al. [20]
33/F	1	2006	Urban	NS	Positive	Skin	Yes	Liposomal AmB, ITRA	3 mg/kg per day 400 mg/day	Death	Therby et al. [21]
60/F	1	2006	Rural	Farmer	Negative	Skin, Bone	Yes	AmB, Terbinafine	1mgkg/day for 30 days 250 mg/day	Favorable clinical response at months 8	Ngatse-Oko et al. [22]
41/M	1	2006	Urban	NS	Positive	Skin	Yes	AmB ITRA	NS	Favorable outcome. No relapse at 6.5 years.	Breton et al. [23]
15/M	1	2010	Urban	NS	Negative	Skin, Lung	Yes	None	-	Death	Okoko et al. [24]
1/M	1	2011	Urban	-	Negative	Eye	No	AmB	0.5 mg/kg Given as intravenous	Favorable outcome at months 6	Eboulabeka et al. [25]
9/F	1	2017	Urban	NS	Negative	Skin	Yes	KETO	200 mg × 2 /days	Insidious outcome at month 1. Death at months 3	Babela et al [26]
3/M	1	2017	Urban	-	Negative	Skin	Yes	ITRA	200 mg/day in per os	Insidious outcome at day 21. Death at months 2	
4/M	1	2017	Urban	-	Negative	Bone	Yes	ITRA	200 mg/day in per os	Insidious outcome at day 28. Death at months 3	
7/F	1	2014	Urban	NS	Negative	Skin, Bone	Yes	Surgery, ITRA	400 mg × 2/days. Given orally	Favorable clinical response at months 3	Paugam et al. [27]
30/F	1	2019	Urban	Unemployed	Negative	Skin	No	Surgery, ITRA	800 mg/days for 12 weeks	Favorable outcome	Boukassa et al. [28]
29/F	1	2019	Urban	Secretary	Negative	Skin	No	Surgery, ITRA	800 mg/days for 12 weeks		
60/M	1	2019	Urban	Administrator	Negative	Skin, lung	Yes	Surgery, ITRA	800 mg/days for 12 weeks	Death	
52/M	1	2019	Urban	Construction worker	Negative	Skin, lung	Yes	Surgery, ITRA	800 mg/days for 12 weeks	Death	
34/M	1	2019	Urban	Sawyer	Negative	Skin, lung	Yes	None	-	Death	
30/F	1	2018	Urban	NS	Negative	Skin, bone	Yes	ITRA	800 mg/days for 12 weeks	Favorable outcome	Boukassa et al. [29]

AmB, Amphotericin B; ITRA, Itraconazole; KETO, Ketoconazole; NS, not specified; NT, not tested.

Urban referred to the capital city, Brazzaville.

**Table 2:** Description of 3 cases of *Hcc* infection in the RoC.

Age/ Sex	No. of cases	Years	Origin areas	Occupation	HIV infection	Site of infection	Disseminated form	Treatment	Posology	Outcome	References
49/M	1	1978	Urban	NS	NT	Mouth, liver	Yes	AmB	NS	Lost to follow-up	Lapèze et al. [30]
22/F	1	1984	Urban	NS	Negative	Skin	Yes	AmB	NS	Death	Carme et al. [31]
46/M	1	1991	Urban	NS	Positive	Skin	Yes	AmB	NS	Favorable outcome	Jaussaud et al. [32]

AmB, Amphotericin B; NS, not specified; NT, not tested; RoC, Republic of Congo.

Of the 57 cases of histoplasmosis, we found males (*n =* 30; 52.6%) to be as frequently infected as females (*n* = 14; 24.6%) with the sex ratio (M/F) at 2.1. The mean age and the median age were calculated at 24 ± 17.6 and 22 years, respectively. The patient’s age ranged between 13 months and 60 years.

An equal number of cases were observed in adults in the third or fourth decades (*n =* 14; 24.6%) and in children aged ≤15 years (Tables 1 and 2). Eight patients were under 10 years. The HIV testing results were mentioned for 7 children and none were HIV–positive. In 10 children, *Hcd* infection was disseminated.

Of the 54 *Hcd* cases, 2 cases were diagnosed in Congolese (from the RoC) expatriates living in France, a man arrived 15 years previously [23] and a woman for some months [21]. A single case of *Hcd*-disseminated infection was diagnosed in the RoC in a foreign national, a Chadian man living in the RoC for an indeterminate time [10]. In contrast, 51 (94.4%) of the 54 cases were definitely acquired in the RoC, as these patients had never traveled outside the country. No documented exposure to caves was reported in those cases and no clustering of cases was evident. Only 5 cases (9.3%) reported being farmers as their occupation in our series. The result for HIV testing in 54 cases of *Hcd* infection is mentioned for 39 patients of whom 20 were HIV–infected (51.3%). In adults, 9 cases (15.8%) of *Hcd* infection were associated with HIV/AIDS. In 38 cases (70.4%), patients had *Hcd*-disseminated infection indicated by infection of at least 2 noncontiguous body sites.

We found 3 cases (5.3%) of *Hcc*-disseminated infection out of the 57 cases. From these, 2 cases were diagnosed in patients of foreign nationality living in the RoC [30,31] and 1 case in a Congolese (from the RoC) patient living in France [32]. One of those cases had a disseminated infection in the context of HIV infection [30]. The first case was diagnosed in 1984 in a 22-year-old young Zairean woman (people of the Democratic Republic of Congo, formerly Belgian Congo or Zaïre) living in the RoC for an indeterminate period [31]. The second case was reported in 1991 in a Congolese AIDS patient living in France with an infection suspected to be acquired in Central Africa [32]. In the last case, Lapèze and colleagues reported a case of *Hcc*-disseminated infection occurring in a French patient living in the RoC for 3 years with a history of traveling and living in Indonesia (6 years), Chad (6 years), and Gabon (2 years).

**Fig 2:**
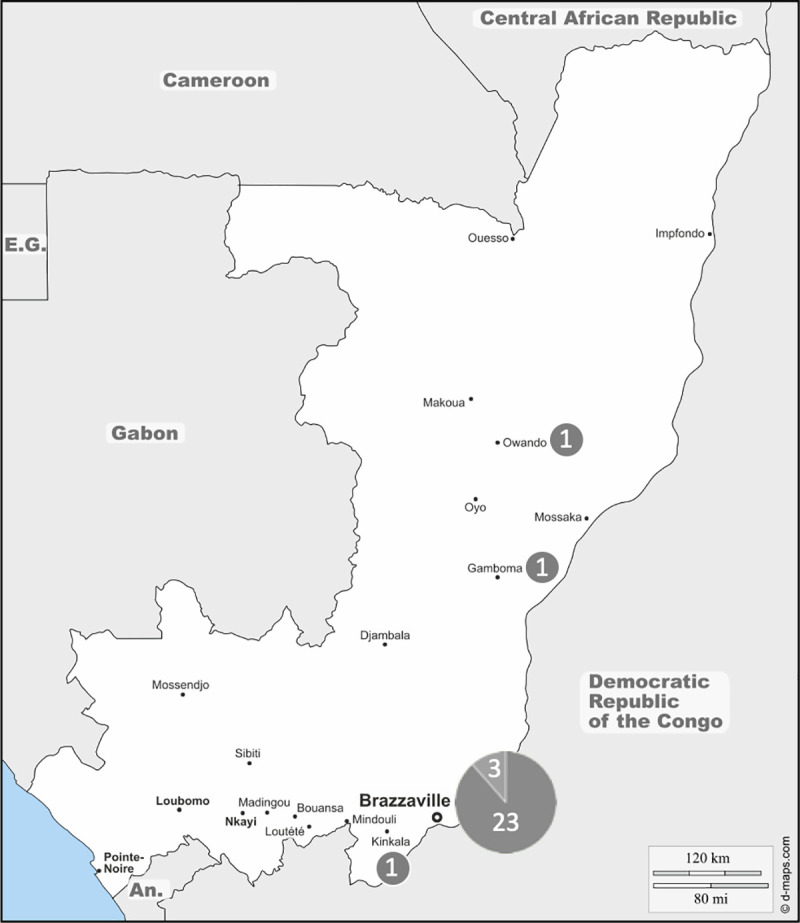
Distribution of reported cases of histoplasmosis in the RoC (1954–2019). Note that the size of the pastilles is not correlated with the number of cases indicated within. (https://d-maps.com/carte.php?num_car=3378&lang=fr.) Dark gray: *Histoplasma capsulatum* var. *duboisii*. Light gray: *Histoplasma capsulatum* var. *capsulatum*.

Although there is no particular regional distribution, the possible origin of infection was notified for 29 patients (50.9%) out of the 57 cases of histoplasmosis reported in the RoC. Patients originated from urban areas (Brazzaville, the capital city) in 26 cases (45.6%) and only 3 cases have mentioned the rural origin of patients’ regions (Gamboma, Owando, Kinkala). Further related areas of rural origin were not mentioned in 28 cases (49.1%) and these cases were excluded from the map (Fig 2).

#### Clinical presentation

The clinical features of the 54 cases of *Hcd* infection are presented in Table 3. Skin lesions (46.3%) including cutaneous and subcutaneous lesions, lymphadenopathy or lymph nodes (37%), and bone lesions (26%) were the most reported clinical presentations. As the most frequent type of lesion, cutaneous lesions resemble *Molluscum contagiosum* (15%) and usually take the form of umbilicated papules or nodules on the skin of limbs and face. Liver and spleen involvement was reported in 5 cases (9.3%). Lung and mucosal infection were reported with equal frequency (7.4%). Of the 14 pediatric cases of *Hcd* infection, the patients presented with bone lesions in 3 cases [14] of which 2 patients had major bone damage [16,27]. On X-ray, the images of bone lysis are observed, often resulting in the form of poorly defined bone cyst.

**Table 3:** Clinical presentation in 54 cases of *Hcd* infection in the RoC.

Sex ratio (M/F)	2.2	
Mean age ± SD	23 ± 17.4	
Clinical manifestations	No. of cases	%
Skin lesions	25	46.3
Lymph nodes	20	37
Bone lesions	14	25.9
*Molluscum contagiosum*-like lesions	8	14.8
Liver/spleen involvement	5	9.3
Mucosal lesion	4	7.4
Lung disease	4	7.4
Adrenal enlargement	1	1.9

#### Laboratory diagnosis

**Fig 3:**
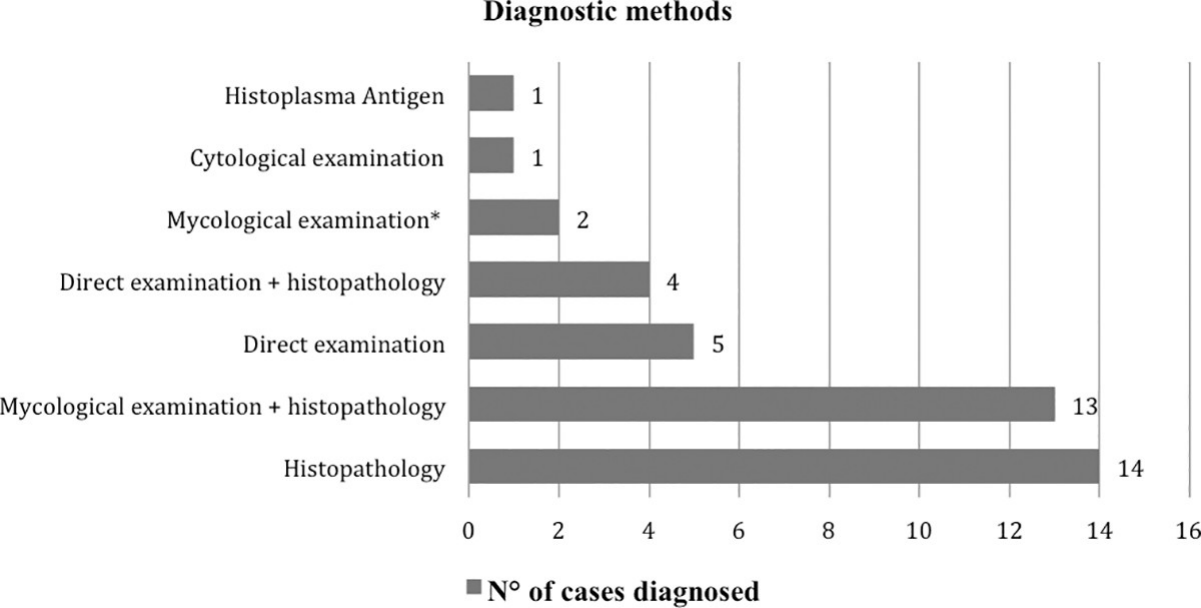
Diagnostic methods used for the diagnosis of *Hcd* cases in the RoC since 1954. Mycological examination* = Direct examination and culture.

**Fig 4:**
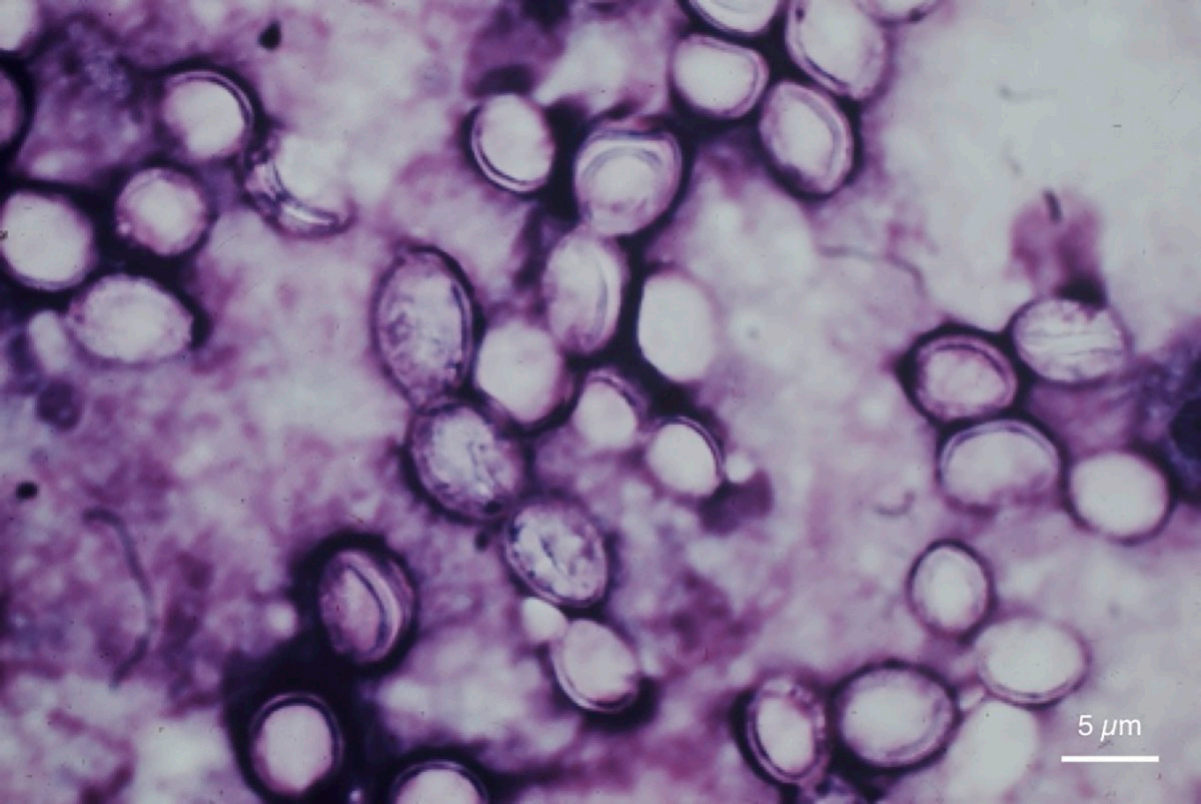
Direct examination of pus from a mesenteric adenomegaly showing large yeast characterized by a large budding site (Giemsa staining, original magnification ×400).

The diagnostic methods of *Hcd* infection are summarized in Fig 3. Mostly diagnosis was based on histopathology examination of biopsy specimen (25.9%) and mycological examination (Fig 4) of different fluids or skin scrapings in combination with histopathology (24.1%). A positive *Histoplasma* antigen was reported in a single case of *Hcd*-disseminated infection diagnosed, in France, in an HIV–positive woman originated from the RoC [21]. Diagnosis of *Hcc* infection was based on direct examination of mucopurulent serosities in 2 cases out of 3. Bone marrow revealed histiocytes with small oval elements surrounded by a clear halo in a single case of a *Hcc*-disseminated infection in an HIV–negative foreign woman living in the RoC [31].

#### Treatment

Treatment information was described in 40 (70.2%) of 57 cases of histoplasmosis. Twenty-six patients (65%) were treated with intravenous amphotericin B (AmB) as first-line therapy. The use of itraconazole (ITRA) was reported only in cases of *Hcd* infection (*n =* 10) while 5 patients were treated with ketoconazole (KETO). In 6 cases of *Hcd* infection, the antifungal drug was combined with surgical treatment for example excision of necrosis and paravertebral and epidural abscesses. Terbinafine was used as a maintenance therapy after AmB in 1 case of *Hcd* infection with bone lesion [22]. All patients presented with *Hcc* infection (*n =* 3) were treated with AmB. Two patients did not receive any antifungal treatment, either due to the misdiagnosis of the disease in favor of tuberculosis or delayed diagnosis of a severe case leading to the rapid death of the patient.

Clinical outcome depended on a number of factors, such as the type of disease (*Hcd* or *Hcc* infection), early or prompt diagnosis, and accessibility to the effective drugs. Most cases with skin lesions (*n =* 20) including those with cutaneous and subcutaneous lesions and disseminated diseases (*n* = 10) resolved with AmB and ITRA, while response to KETO was poor leading to the death of patients (*n* = 4).

## Discussion

Human histoplasmosis is due to 2 different fungal varieties in which geographical distribution differs, and that lead to different clinical presentations. However, molecular analysis reveals that strains of both varieties isolated in Africa belong to the same clade, suggesting that the orientation toward one or the other is driven by environmental conditions or mode of contamination rather than by intrinsic genetic characteristics [33]. To the best of our knowledge, no animal model of *Hcd* infection has yet been set up, that could support different virulence between the varieties. A major result of our study is the large dominance of *Hcd* infection in contrast to *Hcc* infection in the RoC. The first case of *Hcd* has been described in 1952 by Dubois and colleagues in a European man coming from the Democratic Republic of Congo (formerly, Belgian Congo or Zaïre), a border country of the RoC [10,34].

In regard to the total population of the country (5.2 million inhabitants), the number of *Hcd* infection (54 cases) gives a higher prevalence of this infection, unlike other countries in central Africa [5]. Considering the difficulties in diagnosis in our country, it is likely that this prevalence is underestimated. In contrast, the number of *Hcc* was few, as only 3 cases of uncertain geographical origin have been described from the RoC. In addition to a limitation in the availability of the most key diagnostic tools, one can imagine particular geoclimatic local conditions can play a role. Indeed, a remarkable annual rainfall, a high degree of humidity, and a reduced variation in diurnal temperature characterize the RoC climate. We did not observe any particular geographical distribution within the country in the cases reviewed. The possible source of infection was notified for 29 patients (50.9%), and a rural origin of the patients was only notified for 3. Whether or not local environments favor the “expression” of *Hcd* infection rather than *Hcc* infection required further studies. Finally, it should also be mentioned that facilities in the diagnosis of histoplasmosis are limited mainly to Brazzaville, the capital and largest city. Indeed, most of infected patients were from urban areas (Brazzaville) but there might be a bias because the population is predominantly urban (62.2%), and 56.5% of the total population lives in Brazzaville [35]. Also, the diagnosis can only be achieved in medical centers from urban areas. This contrasts with previous reports suggesting that *Hcd* infection is mainly a rural mycosis in other countries [26,36]. However, the increase in HIV–positive patients in the RoC probably promotes cases of histoplasmosis being seen in Brazzaville. Nevertheless, it should be noted that in central Africa, Zaïre was the main focus of *Hcd* infection with 26 cases. In contrast, all of the cases (*n =* 7) described before 1984 originated from a rural area (the plateaux and Pool region) of the RoC [15]. Although 5 cases were reported in farmers, the overall risk of *Hcd* infection is not well understood. Moreover, Carme and colleagues [14] reported that most patients with *Hcd* infection are farmers, most consistent with a soil-related acquisition. In the study from Democratic Republic of the Congo, which shares a border with the RoC, Pakasa and colleagues reported that 4 patients infected with *Hcd* had collected guano from bat roosts to fertilize gardens [37]. In the environment, *Histoplasma* may be found in so-called microfoci in endemic areas for histoplasmosis. The main characteristic of these microfoci is contamination with bird/or bat guano, notably caves, but the extent of its natural occurrence remains largely unexplored. Of note, in a recent review, no difference was found between the 2 varieties in terms of environmental and wilderness-related risk factors [38].

Interestingly, only 3 cases of *Hcc* infection were recorded and none of these cases seemed to be acquired in the RoC. Additional studies are warranted to explore more precisely the conditions leading to infection with one variety rather than the other.

Moreover, 14 cases of *Hcd* infection were recorded in children and none of them was HIV–positive. This finding agrees with that of previous reports [18,24–26]. A review of the risk factors by Lopez and colleagues revealed that environmental fungal exposure was the most important contributing risk factors to the acquisition of *Hcd* infection in children [39].

In *Hcd* infection cases from the RoC, skin lesion including cutaneous or subcutaneous and mucosa involvement and lymph nodes were the most common as noted by Carme and colleagues [14] and Develoux and colleagues [5]. The clinical manifestations of *Hcd* infection are more commonly the skin, subcutaneous tissues, lymph nodes and bones unlike *Hcc* infection which generally involves lungs and mucosa and rarely shows cutaneous or bone lesions. The cutaneous manifestations in *Hcd* infection are papular, nodular, ulcerative, eczematoid lesions [40]. However, the severity of the disease is related to the immunity status of patients. Nine *Hcd* infection cases (51.3%) in adults were associated with HIV/AIDS in the RoC. Similarly, Oladele and colleagues reported that the disease manifestations vary depending on immune status of patients, the number of fungal particles inhaled (particularly for *Hcc*), and the virulence of the infective strain [4]. A single RoC case of *Hcc*-disseminated infection was reported with a painful oral lesion on a background of leukoplakic mucosa [30].

Oral and laryngeal lesions are possible with *Hcc* infection. Most oral lesions occur in the *Hcc*-disseminated form of the disease and may affect any area of the oral cavity but are most commonly found on the tongue, palate, and buccal mucosa [41,42]. Patients may present with painful ulcers that persist for several weeks [30,41]. *Hcd*-disseminated forms (51.3%) were not frequently reported in HIV–infected patients. In the current decades, the rate of disseminated forms (60.6%) has significantly increased without correlation with HIV infection [5]. The bone lesions in *Hcd* infection are of a lytic type found at cranial, maxillary, femoral, tibial, and spinal bones where they simulated Pott disease (tuberculous osteomyelitis) [28]. All organs and tissues can be involved [43]; indeed, we found that skin, mucosa, bone, and lung were mostly involved in the *Hcd* infection.

The WHO-endorsed essential diagnostic tests (2019) for fungal diseases are direct microscopy and histopathology, fungal culture, blood culture, and *Histoplasma* antigen [44]. In the RoC, most of the reported cases were diagnosed by histopathology [20,21,45] and mycological examination (direct microscopy and culture) [14,21]. It is important to recall that due to the coexistence of the two *H*. *capsulatum* varieties in Africa, differential diagnosis cannot be made only based on culture, as it is similar for both. Only the demonstration of the ovoid budding yeast (Fig 4) with thick cell walls and much larger (6 to 12 μm in diameter) and intracellular fat droplets supports the identification of *Hcd* [46]. In addition, *Hcc* appears as a 2 to 4-μm narrow-based budding yeast on histopathology of a tissue specimen [46]. Thus, the histological appearances of *Hcc* must be differentiated from other microorganisms, principally *Candida glabrata*, *Cryptococcus neoformans*, *Leishmania* spp., *Blastomyces dermatitis*, *Pneumocystis jirovecii*, *Talaromyces (*formerly *Penicillium) marneffei*, *Toxoplasma gondii*, *and Trypanosoma cruz* [46]. Although, many of these organisms are not endemic and would only be seen in travelers abroad. Mostly, the characteristics that help distinguish these yeasts include predominant cellular location (intracellular for *H*. *capsulatum*, predominantly extracellular for *C*. *glabrata*), shape and size variation (uniform versus heterogenous), histopathologic response (granulomatous versus suppurative), and culture. Also, the use of specific histochemical stains such as Giemsa, hematoxylin and eosin, Gomori methenamine silver, and periodic acid-Schiff facilitates the differentiation of these pathogens [46]. Detection of *Hcc* is also possible in approximately 40% of blood smears of patients with disseminated histoplasmosis [43–45], though this was not yet reported in the RoC. While antigen detection and PCR emerge as valuable adjunctive diagnostic tools, those are not yet available in the RoC.

The treatment of histoplamosis is a challenge for physicians in most low- and middle-income countries including the RoC, where WHO-listed essential systemic antifungal drugs namely itraconazole, voriconazole, flucytosine, and AmB are not always available. Currently, only fluconazole, ketoconazole (withdrawn across most of the world because of toxicity), topical miconazole, griseofulvin, and nystatin are available in the RoC.

In our review, most patients were treated with intravenous AmB. The lack of itraconazole availability is a serious limitation as only fluconazole can be given for maintenance therapy in the RoC. Fluconazole has a lower efficacy compared to itraconazole in the treatment of histoplasmosis due to its lower bone penetration and lower intrinsic activity [47]. Symptoms of *Hcd* infection in children are similar to those that occur in adults, with some exceptions, thus treatment recommended for adults is considered for children.

## Conclusions

Our study shows that histoplasmosis appears to have a higher rate of prevalence in the RoC compared with other countries in central Africa. Surprisingly, 94.7% of the cases were due to *Hcd*, and *Hcc* cases may have been infected out of the RoC. While both varieties cannot be distinguished based on molecular analysis, there is no clear explanation for the reasons leading infection with one or other. Currently the differentiation between the two varieties only relies on direct examination of biopsy specimens or pathologic fluids, and set-up of more standardized techniques allow this differentiation is desirable. This review also highlighted the importance of pediatric cases of histoplasmosis due to *Hcd*. This observation should also drive future studies to better understand the mode of contamination, which remains uncertain.

While most of the patients were treated with intravenous AmB, itraconazole should be more widely available in Africa as it represents a valuable alternative for ambulatory treatment.
